# A multi-functional bubble-based microfluidic system

**DOI:** 10.1038/srep09942

**Published:** 2015-04-23

**Authors:** Khashayar Khoshmanesh, Abdullah Almansouri, Hamad Albloushi, Pyshar Yi, Rebecca Soffe, Kourosh Kalantar-zadeh

**Affiliations:** 1 School of Electrical and Computer Engineering, RMIT University, Melbourne, Victoria 3001, Australia

## Abstract

Recently, the bubble-based systems have offered a new paradigm in microfluidics. Gas bubbles are highly flexible, controllable and barely mix with liquids, and thus can be used for the creation of reconfigurable microfluidic systems. In this work, a hydrodynamically actuated bubble-based microfluidic system is introduced. This system enables the precise movement of air bubbles *via* axillary feeder channels to alter the geometry of the main channel and consequently the flow characteristics of the system. Mixing of neighbouring streams is demonstrated by oscillating the bubble at desired displacements and frequencies. Flow control is achieved by pushing the bubble to partially or fully close the main channel. Patterning of suspended particles is also demonstrated by creating a large bubble along the sidewalls. Rigorous analytical and numerical calculations are presented to describe the operation of the system. The examples presented in this paper highlight the versatility of the developed bubble-based actuator for a variety of applications; thus providing a vision that can be expanded for future highly reconfigurable microfluidics.

Microfluidic systems with integrated mechanical, electrical, and optical components facilitate conducting comprehensive assays for various applications in biology^1^,^2^,^3^,^4^,^5^, advanced clinical procedures^6^,^7^,^8^,^9^, and chemistry^10^,^11^. In particular, incorporating components such as micropumps, microvalves, micromixers and microactuators lend versatility to microfluidic systems for the ultimate realisation of all-in-one units^12^.

Air bubbles can significantly impact the behaviour of flow within microfluidic systems^13^,^14^,^15^. In general, unwanted bubbles pose a challenge for the operation of microfluidic platforms, and different approaches have been devised to remove or prevent the formation of the bubbles^16^,^17^,^18^. However, bubbles can be very well used to our advantage in microfluidic systems. Controllability, cleanliness, compressibility, and bio-compatibility of air bubbles have inspired researchers to develop a variety of innovative bubble-based components such as micropumps^19^,^20^,^21^, micromixers^22^,^23^,^24^, microvalves^20^,^25^,^26^,^27^, and microactuators^28^,^29^.

Several strategies have been used for actuating gas bubbles within microfluidic systems. This includes the application of laser pulses for the local generation of gas bubbles. The rapid expansion of such bubbles can be efficiently utilised for pumping^19^,^20^, mixing^22^ and controlling the flow^20^, releasing of trapped particles^28^ or deflecting moving particles^29^. Despite its high controllability, the implementation of laser pulses can rapidly increase the local temperature and potentially damage suspended organic materials, rendering the system problematic for bio applications^30^,^31^. Alternatively, the generation of gas bubbles has been achieved by electrolysis using inert electrodes such as platinum^21^. The volume of the bubbles, which depends on the current applied to the electrodes can be periodically changed to pump the liquid through the main channel^21^. Obviously, the limitation of such systems is their dependence on ionic liquid.

Another strategy for implementing air bubbles is to trap them inside the cavities pre-designed within the microfluidic system, and later use them for controlling the liquid flow. For example, the thermal expansion of air pockets trapped within side chambers, by means of resistive heaters, has been utilised for controlling the flow in the main channel^26^. Alternatively, passive expansion of air pockets stored inside the side chambers, due to pressure drop in the main channel, has been demonstrated for the realisation of valve concept^27^. Additionally, the oscillation of air bubbles trapped inside horse-shoe structures, by means of acoustic waves, has been implemented for disturbing streams in a microfluidic channel^23^. Despite simplicity, performance of such systems is subjected to limitations by the possible departure or displacement of the bubbles from their designated locations.

Another strategy is to implement the air bubbles supplied from an external air supply. These air bubbles can be simply generated and controlled by applying a manual vacuum to the outlet of a microfluidic channel^24^. The movement of such air bubbles through a series of bifurcated channels, which are patterned at the downstream of a main channel, has been shown to cause the flow oscillation between the two branches of bifurcated channels and the efficient mixing of neighbouring flows^24^. Alternatively, the pneumatic actuation of air bubbles along the side channels can be utilised to open or close the main channel^25^. The open/close modes can be achieved according to the pressure of the air supply^25^. Despite high controllability and scalability, such a system requires external air supplies and relies on digital pressure controllers.

Despite the versatility and great potentials of the existing bubble-based systems, there are still many opportunities for enhancing their performance^32^. These systems can enjoy more widespread attention and offer a variety of advanced physical, chemical and biomedical applications by incorporating novel bubble actuation strategies, which: (i) do not increase the temperature of the media; (ii) are biocompatible; (iii) require simple fabrication and operational processes; (iv) need minimal interactions with optical, electrical, and mechanical equipment; (v) involve inexpensive, small, and portable components; and (vi) allow for multiple function capabilities (e.g. mixing and controlling of flow together with the manipulation of suspended particles and bio particles).

We introduce a novel strategy for the hydrodynamic actuation of air bubbles within the axillary feeder channels of a microfluidic system. A high-resolution hydrodynamic actuator is fabricated to form a small air bubble at the tip of a tube that is prefilled with water, and move the bubble along the feeder channel. This enables altering the characteristics of the flow within the main channel according to the speed, location and size of the bubble, enabling a variety of applications in microfluidics. We show that the oscillation of the bubble along the feeder channel induces a strong lateral disturbance in the main channel. We also demonstrate that further pushing of the bubble along the feeder channel can partially or fully block the flow in the main channel. More interestingly, creating a large bubble in the main channel changes the flow streamlines, enabling the hydrodynamic patterning of suspended particles. Altogether, we present approaches for creating highly controllable multi-functional bubble-based systems that can be reconfigured for various microfluidic applications.

## Results and Discussions

### Changing the geometry of the microfluidic channel utilising bubbles

**Figure 1:**
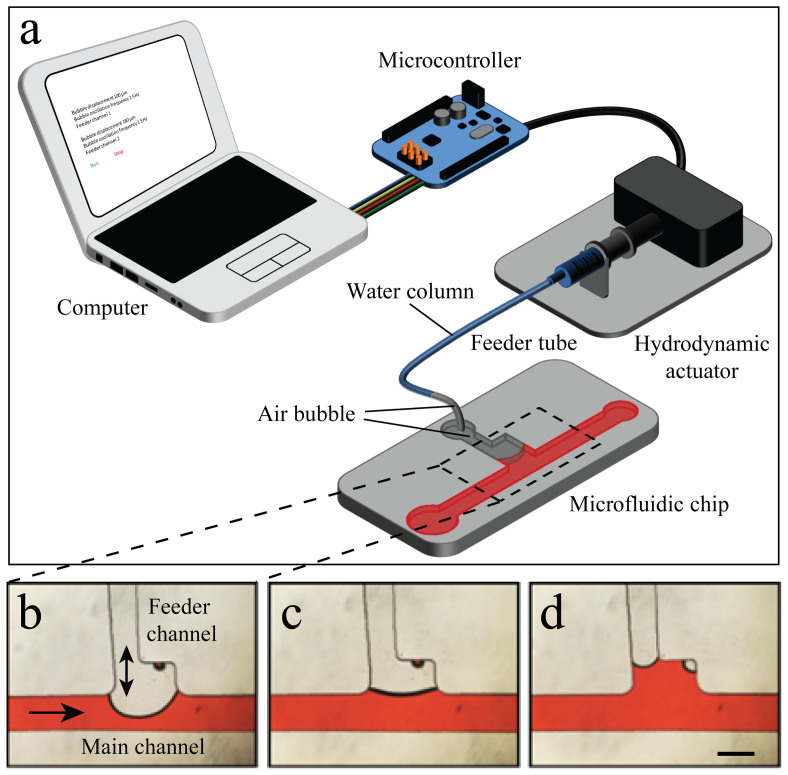
Microfluidic-based bubble actuator: (a) Schematics of the actuator. Moving the air bubble in the feeder channel changes the configuration of the main channel and leads to creation of (b) narrowed channel, (c) straight channel or (d) cavity (side-well). Scale bar is 500 µm.

Figure 1a shows the schematics of the microfluidic-based bubble actuator, which consists of a computer, a microcontroller, a hydrodynamic actuator and a microfluidic chip. The hydrodynamic actuator is the heart of the system, which consists of a high-resolution stepper-motor, a custom-made mechanical gear to transform rotational motion into linear motion, and a glass syringe, which is connected to the microfluidic chip *via* a tube, which we call the feeder tube, as further detailed in Supplementary Information-1. The microfluidic chip consists of one or several inlet reservoirs to dispense the liquids, a main channel, and an outlet port, which is connected to an external syringe pump, and is operated in withdrawal mode to provide desired flow rates through the main channel. Moreover, the microfluidic chip has a feeder channel, which is connected to the hydrodynamic actuator *via* the feeder tube (Figure 1a).

To operate the hydrodynamic actuator, the glass syringe and feeder tube are manually filled with water while the microfluidic chip is filled with the desired working liquid. The syringe is then connected to the mechanical gear and pulled 50 steps to draw a small air pocket with a volume of ∼ 1.6 μL at the tip of the feeder tube. The syringe is then pushed to drive the air pocket into the feeder channel, while a large portion of the air pocket still remains in the feeder tube. For example, when using a feeder tube with an inner diameter of 1/32 inch the length of the air column inside the feeder tube is ∼ 1.5 mm.

The air pocket is surrounded by the water column inside the feeder tube on one end and by the liquid moving inside the main channel of the microfluidic chip on the other end, and therefore can be referred to as an “air bubble”. The characteristics of the flow inside the main channel can be influenced according to the location of the bubble inside the feeder channel. For example, pushing of the bubble through the feeder channel narrows down the main channel (Figure 1b), pushing the bubble right to the outer border of the junction maintains the straight geometry of the main channel (Figure 1c), whereas locating the bubble just at the inner border of the feeder channel junction creates a cavity along the sidewall of the main channel (Figure 1d).

The water column and the air bubble are in series, and neglecting the compressibility of the air bubble due to small pressure changes, the displacement of the water column is directly transferred into the displacement the air bubble inside the feeder channel, enabling the hydrodynamic actuation of the air bubble. To minimise any discrepancies associated with the elasticity of the syringe or tube, we apply a glass syringe in conjunction with a thick feeder tube (inner diameter = 1/16 inch, outer diameter = 3/32 inch). The minimum volume (*V*_min_) of the water displaced by the hydrodynamic actuator can be calculated by multiplying the surface area of the syringe by the translation of the mechanical gear per each step, 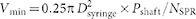, where *D*_syringe_ is the internal diameter of the syringe, *P*_shaft_ is the linear pitch of the mechanical gear, and *N*_SPR_ is the number of steps per revolution of stepper motor. Considering the specifications of our system, *V*_min_ is obtained as 22 nL/step, as detailed in the Methods section, enabling the precise movement of the air bubble inside the feeder channel. The operation of the hydrodynamic actuator is demonstrated in Supplementary Movie 1.

In order to push the bubble through the feeder channel, the pressure inside the bubble should be slightly higher than that of the liquid at the feeder channel junction, which can be expressed as:
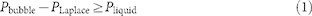
where *P*_Laplace_ is the pressure jump at the interface of the bubble and liquid. Considering that the vertical and horizontal curvatures of the bubble are bounded by the height and width of the feeder channel (*r_z_∼ H*_feeder_*/*2*,* *r_xy_∼* *W*_feeder_*/*2), this pressure jump can be approximated as:
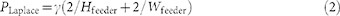
where *γ* is the interfacial tension which for the case of an air bubble in water is 0.075 N/m. The local pressure of the liquid at the feeder channel junction can be calculated using the modified Hagen–Poiseuille equation for rectangular channels^33^,^34^ as:
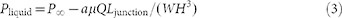
where *P*_∞_ is the ambient pressure, *µ* is the dynamic viscosity of the liquid, *Q* is the flow rate of the liquid, *L*_junction_ is the distance between the feeder channel junction and the inlet reservoir, *W* and *H* are the width and height of the main channel, respectively, and *a* is a dimensionless number that depends on the aspect ratio of the channel, which can be calculated as 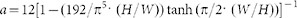
33,34. To be more accurate, the pressure drop along the channel can be multiplied by *k*_contraction_, to take into account the sudden pressure drop occurring at the interface of the inlet reservoir and the inlet channel^35^.

### Bubble-based micromixer

**Figure 2:**
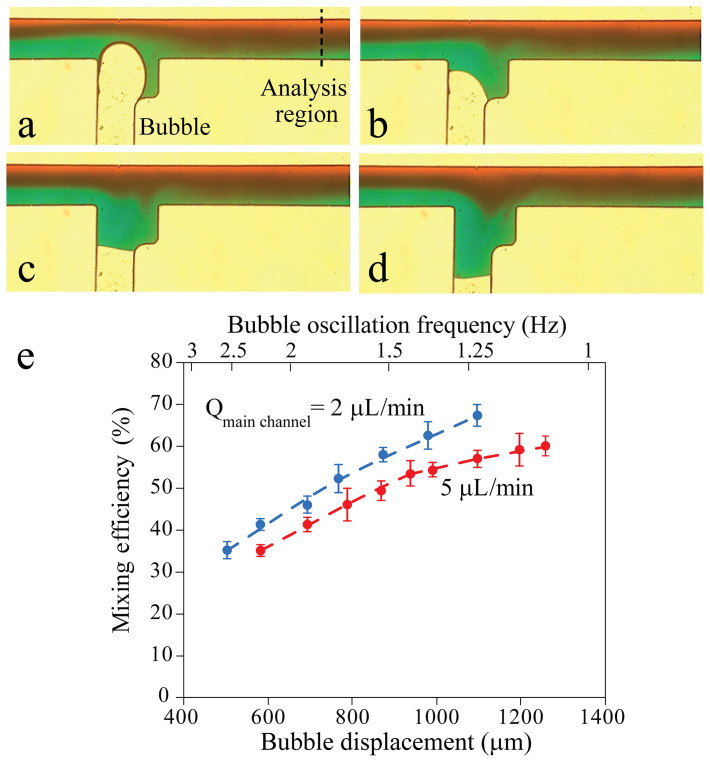
Active mixing of two neighbouring fluids moving through the main channel using the bubble-based micromixer: (a-d) Show the mixing within one complete cycle of bubble oscillation along the feeder channel, obtained by oscillating the bubble with a displacement of 1095 μm and a frequency of 1.22 Hz with a flow rate of 2 μL/min through the main channel. Scale bar 500 μm. (e) Variation of mixing efficiency with respect to the displacement of the bubble, and the flow rate of the liquid in the main channel. The experiments are repeated at least 5 times and the error bars represent the standard deviation.

Oscillating the bubble inside the axillary feeder channel can effectively disturb the liquid flowing through the main channel. This can lead to a bubble-based micromixer, as shown in Figure 2**.** In this example, the main channel has a width of 500 µm while the feeder channel has a width of 450 µm which increases to 750 μm at the junction. The expansion of the feeder channel at the junction decreases the displacement of the bubble in the vicinity of the junction as given in Methods section, and enables more control over the progress of the bubble inside the main channel. Moreover, it increases the surface area of the oscillating bubble, enhancing the mixing efficiency of the system. A flow rate of 2 μL/min is withdrawn through the outlet of the main channel using a syringe pump. The bubble is oscillated at a displacement of 1095 μm and with a frequency of 1.22 Hz. Water-based food dyes are used in order to distinguish between the neighbouring liquid streams. The mixing is monitored using an inverted microscope equipped with a camera to capture colourful images (see Methods section for detail).

The mixing efficiency is estimated by measuring the colour intensity along a line, which is 2000 µm away from the feeder channel junction (Figure 2a), using the following equation:
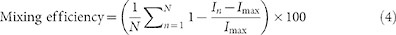
where *N* is the total number of pixels and *I_n_* is the colour intensity for the pixel *n* and *I*_max_ is the colour intensity of the fully mixed region, taken from the interface of the unmixed streams upstream of the feeder channel. Using this equation, a mixing efficiency of 67.2 ± 3% is obtained at the prescribed conditions.

The mixing performance of the system is characterised against different bubble displacements and flow rates through the main channel (Figure 2e). To facilitate the comparison, the average velocity of bubble oscillation along the feeder channel (bubble displacement × frequency of bubble oscillation) is set to 1250 ± 60 µm/s. This means that for larger bubble displacements the frequency of the oscillation is reduced. According to our observations, increasing the bubble displacement enhances the mixing efficiency of the system. For example, at a flow rate of 2 μL/min, increasing the bubble displacement from 767 to 1095 μm leads to enhancing the mixing efficiency from 51.9 ± 4% to 67.2 ± 3% (see Supplementary Movie 2). This is because increasing the bubble displacement augments the lateral displacement of the flowing liquid, and induces more disturbances into it. At the same time, the frequency of bubble oscillation is reduced from 1.56 to 1.2 Hz and thus the flowing liquid has more time to be agitated. In contrast, increasing the flow rate of the main channel reduces the mixing efficiency. For example, at a bubble displacement of 1095 μm, increasing the flow rate from 2 to 5 μL/min leads to reducing the mixing efficiency from 67.2 ± 3% to 58.8 ± 2%, as the ratio of the lateral to axial velocity decreases.

In order to measure the response time of the bubble, we compare the variations of the sinusoidal actuation signal applied to the mechanical gear with the displacement of the bubble tip along the feeder channel when oscillated at a displacement of 1095 μm with a frequency of 1.22 Hz. In doing so, we monitor the displacement of the bubble using an inverted microscope equipped with an electron-multiplying charge-coupled device (CCD) camera, which is capable of acquiring images at a rate of 50 frames per second. The images are post-processed using MATLAB’s image processing toolbox to track the tip of the bubble (detail in the Methods section). Supplementary Information-2 shows the normalised variations of the applied signal and the displacement of the bubble tip with respect to time in 3 cycles. The results indicate a short delay of < 20 ms when the bubble is moving towards the sidewall with an elongated delay of up to 80 ms when the bubble is moving away from the sidewall.

The stability of the oscillating bubble depends on the location of the bubble tip with respect to the main channel. For example, for the case of Figure 2a–e, the initial location and displacement of the bubble is chosen such that the bubble tip can penetrate up to 200 µm inside the main channel. In this case, the bubble does not block the main channel and can be operated without detachment from the junction even at a high flow rate of 17.5 µL/min, although the mixing efficiency substantially reduces at such a flow rate. Alternatively, the initial location and displacement of the bubble can be adjusted to keep the bubble tip inside the feeder channel (**see** Supplementary Movie 3). In this case, the bubble maintains its stability and harmonic oscillation even at a high flow rate of ∼ 27 µL/min. The mixing efficiency of the system can be improved by increasing the number of feeder channels along the main channel. Several bubbles can be actuated using the same mechanical gear without complicating the operational procedure of the system.

In addition, the capability of our bubble-based micromixer to disturb liquids in smaller microfluidic systems was examined by reducing the width of the main channel to 200 µm, as presented in Supplementary Information 3.

### Bubble-based microvalve

**Figure 3:**
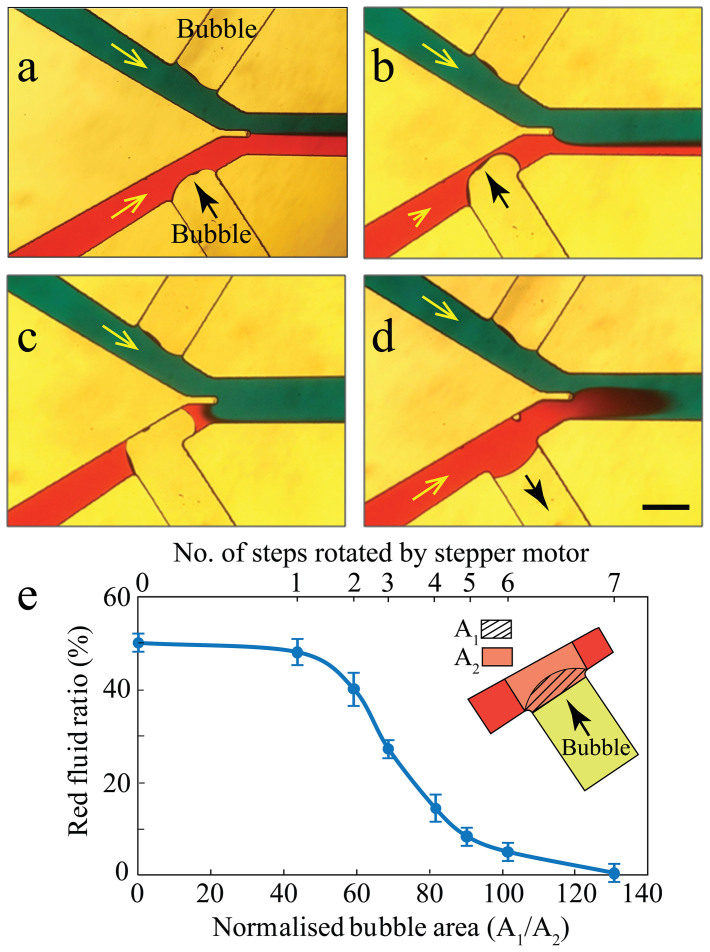
Different operational modes of the bubble-based microvalve under a flow rate of 10 μL/min withdrawn from the outlet of the main channel: (a) Operation in the fully open mode with equal flows of red and blue streams through the main channel, (b) Operation in the partially closed mode with the flow of red stream reduced to half, (c) Operation in the fully closed mode with no red stream flowing through the main channel, (d) Re-opening the microvalve to retain the flow of red stream through the main channel. Scale bar is 500 μm. (e) The variations of the red fluid ratio with respect to number of steps rotated by the stepper motor, and the area covered by the bubble. The experiments are repeated at least 4 times and the error bars represent the standard deviations.

Using a Y-shaped channel and connecting the axillary feeder channel to one or both inlets, the system can be used as a microvalve. The size of the bubble at the feeder channel can be adjusted to obtain fully open, partially closed and fully closed modes, as shown in Figure 3. In this experiment, the two inlet channels have a width of 350 μm, the two feeder channels have equal widths of 600 μm, and the main channel has a width of 500 μm. A flow rate of 10 μL/min is withdrawn through the outlet of the main channel.

Fully open mode is achieved by maintaining the tip of the air bubble close to the feeder channel junction with minimum disturbance of red stream flowing through the bottom inlet channel. In this mode, the red and blue streams enter the main channel at the same ratio (Figure 3a). Partially closed mode is achieved by further pushing the air bubble into the bottom inlet channel. The expansion of the bubble increases the pressure drop along the inlet channel, and thus reduces the red stream through the channel (Figure 3b). Fully closed mode is achieved by further pushing the bubble into the inlet channel to make an intimate contact with the opposite sidewall of the channel. In this mode, the red stream is completely blocked, and only the blue stream enters the main channel (Figure 3c).

The blocking of the channel leads to an increased pressure difference along the two ends of the bubble, which pushes the bubble towards the main channel, as evidenced by the configuration of the bubble in Figure 3b and 3c. Our experiments indicate that at a flow rate of 10 μL/min, the fully closed mode can be maintained for at least 15 minutes without the bubble being stretched towards the main channel or pinched off. Moreover, after achieving the fully closed mode, the flow rate can be increased to ∼ 24 μL/min without destabilising the bubble. However, further increase of the flow rate can lead to the destabilisation and detachment of the bubble from the feeder channel.

The relation between the stability of the bubble and the flow rate of the liquid applied through the Y-shaped channel is analysed as follows. At the upstream of the bubble, the vertical curvature is bounded by the height of the channel (*r_z_∼* *H*_inlet_*/*2), while the horizontal curvature can be scaled by the width of the channel (*r_xy_∼* *W*_inlet_)^36^, and the pressure jump at the upstream of the bubble can be approximated using 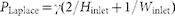. Given that the bubble is pushed by the liquid, the pressure balance at the upstream of the bubble is obtained as:
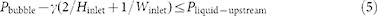
Alternatively, at the downstream of the bubble, the vertical and horizontal curvatures are bounded by the height and width of the channel (*r_z_∼* *H*_inlet_*/*2*,* *r_xy_∼* *W*_inlet_*/*2), and the pressure jump at the downstream of the bubble can be approximated as *P*_Laplace _ = *γ*(2/*H*_inlet _+ 2/*W*_inlet_). Considering that the bubble pushes the liquid, the pressure balance at the downstream of the bubble is obtained as:
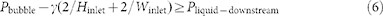
Combining these two equations, the balance between the pressure difference across the bubble (which acts to push and detach the bubble from the feeder channel) and the interfacial pressure (which acts to reserve the bubble at the feeder channel), is obtained as:
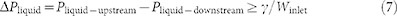
Assuming that the inlet channel is fully closed by the bubble, the pressure at the upstream of the bubble remains as the ambient pressure. Alternatively, the pressure at the downstream of the bubble equals to the pressure at the end of the other inlet channel (just before the Y-junction). Therefore, the pressure difference along the two ends of the bubble can be expressed using: 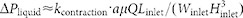. Using this equation and considering *k*_contraction_ = 1.1, the maximum flow rate that the bubble can withstand before being detached from the feeder channel is obtained as 19.8 μL/min, which is close to the flow rate of 24 μL/min obtained in experiments.

The microvalve can be easily switched back to the fully open mode to enable the red stream in the channel (Figure 3d). The developed bubble-based microvalve is opened and closed at least 100 times under the aforementioned conditions, without any significant changes observed in its performance. The variations of the red flow ratio within in the main channel, with respect to the number of stepper motor rotations and the area of the bubble, are shown in Figure 3e. Stepper motor rotations of zero (*N*_steps _ = 0) refers to the case at which the tip of the bubble rests at the feeder channel junction. Our experiments indicate that minor rotation of stepper motor (*N*_steps _≤ 2) does not lead to tangible changes in the red flow, and the system still operates in fully open mode. However, further rotation of stepper motor (2 < *N*_steps_ ≤ 7) leads to quick changes in the red flow, and the system operates in the partially closed mode. Further rotation of stepper motor (*N*_steps_ = 8) leads to blocking of the red flow, and operation in fully closed mode. At the fully closed mode, the area covered by the bubble is 1.28 times larger than the area of the region located at the intersection of the main and inlet channels, as the bubble is pushed towards the main channel (Figure 3c). To improve the response time of the microvalve, narrower feeder channels can be used for enabling the faster movement of the bubble (Supplementary Movie 4).

### Bubble-based microactuator

Moreover, the size of the bubble can be adjusted to enable hydrodynamic patterning of suspended particles through the main channel. Such a patterning is desirable in many flow cytometry investigations^37^. This phenomenon can be achieved when a large bubble is generated in the main channel, leaving a narrow gap, approximately the same size of the suspended particles, between the bubble and the channel walls.

**Figure 4:**
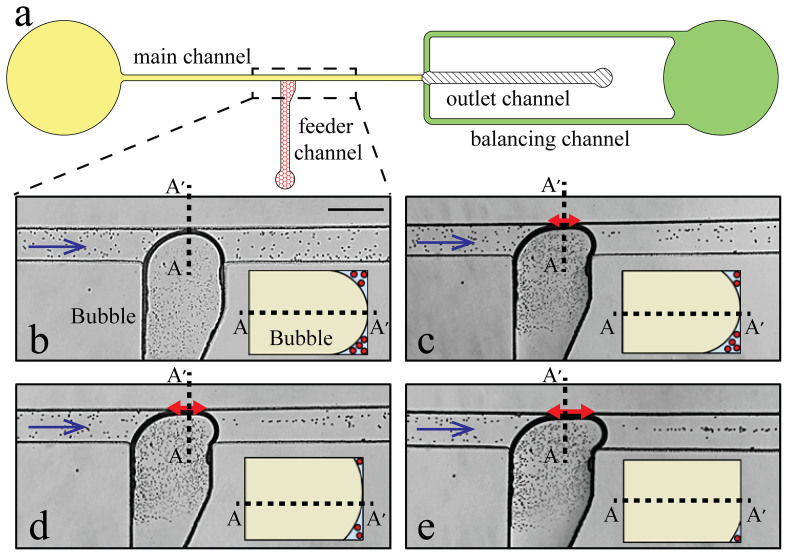
Bubble-based actuator for patterning of suspended 15 µm particles under a flow rate of 2 μL/min withdrawn from the outlet of the main channel: (a) The layout of microfluidic system. The response of 15 μm particles when the bubble is enlarged enough to touch the opposite sidewall of the main channel: (b) The disordered passage of particles through the two gaps formed at the bottom and top corners of the main channel when the bubble tip has just touched the opposite sidewall of the main channel, (c-d) Partial patterning of the particles by further pushing of the bubble, (e) Ordered patterning of the particles along a straight line by further pushing of the bubble. Inset is the schematic cross sectional view of the main channel along AA′ line. The length of the intimate contact between the bubble and the opposite sidewall (shown with red arrow) increases from 0 μm at (b) to 240 μm at (c), to 340 μm at (d) and eventually to 390 μm in (e). Scale bar is 500 μm.

The design of the microfluidic system is changed to maintain the stability and integrity of a large bubble, and avoiding its detachment from the feeder channel, as shown in Figure 4a. The system has two balancing channels to maintain a steady flow at the downstream of the main channel when it is almost blocked by a large bubble. This reduces the pressure on the bubble, enabling the stable performance of the large bubble. The main and balancing channels have a width of 300 μm, while the outlet channel has a width of 600 μm. The feeder channel has a width of 450 μm which linearly increases to 700 µm in the vicinity of the junction (Figure 4a). Unlike Figure 2, in which the immediate widening of the feeder channel does not allow the bubble to fill the entire cross section of the junction in consequential oscillations, the gradual widening of the feeder channel enables the bubble to fill the entire cross section of the junction during the advancement towards the opposite sidewall of the main channel.

Figure 4b–e show the trajectory of suspended 15 μm polystyrene particles (PPS-15.0, Kisker) in the presence of varying bubble sizes when the flow rate of the outlet channel is set to 2 μL/min. The number of the stepper motor rotations required to achieve for each case is measured with respect to the case at which the tip of the bubble rests at the feeder channel junction. For example, the stepper motor is rotated 4 steps to make the tip of the bubble touch the opposite sidewall of the main channel (Figure 4b). This results in a small contact area between the bubble and the sidewall while large gaps at the upper and lower corners of the channel. This allows multiple suspended particles to pass through the gaps with random trajectories, as shown in the inset.

Further rotation of the stepper motor by 1 and 2 steps leads to further pushing of the bubble towards the opposite sidewall, as shown in Figures 4c and 4d, respectively**.** The length of the interface between the bubble and the sidewall, which is shown by red arrows, reaches 240 and 340 µm, respectively. This leads to an increased contact area between the bubble and the opposite sidewall, and in turn smaller gaps at the corners. The trajectories of the particles passing through the gaps are not as random as Figure 4b, but the particles have yet to gain a full order.

By further rotation of the stepper motor by 1 step, the length of the interface between the bubble and the sidewall reaches 390 µm (Figure 4e). A narrow gap about the same size of the 15 μm particles remains between the bubble and the channel walls, as shown in the inset. This allows the passage of one particle at a time, and leads to the formation of a single-particle stream at the downstream of the bubble (see Supplementary Movie 5). Our observations using an inverted microscope indicate that the formed gap can be located at either the bottom or the top corners of the channel.

In order to maintain the stability of the bubble shown in Figure 4e, the pressure difference along the bubble should be counterbalanced by the interfacial pressure, which can be expressed as Δ*P*_liquid_ = γ/*W*_main_ as given in Equation (7). According to lubrication theory, the pressure drop along the bubble can be expressed as follows^36^,^38^:
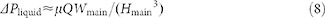
where *ε* is the thickness of the thin film of liquid, which is remained between the bubble and the opposite sidewall. Using this equation, the value of *ε* is obtained as 9.5 µm, which is smaller than the diameter of 15 µm particles. This disparity can be due to the fact that in Equation (8) the pressure drop is calculated by assuming a uniform *ε* along the height of the channel, whereas our observations suggest that the gap forms at either the bottom or top corner of the channel. Moreover, this equation ignores the additional pressure drop due to existence of suspended particles through the gap.

Our extended experiments indicate that the trajectories of particles at the downstream of the bubble depend on their dimensions. For example, when applying a mixed suspension of 3 and 15 µm polystyrene particles to the main channel, we observed that the large particles are patterned along the middle of the main channel, whereas most of the small particles take a different streamline and are patterned very close to the sidewall of the main channel. This response, which is presented in Supplementary Movie 6, suggests the capability of our bubble-based system to sort particles according to their dimensions. In this example, the main channel has a width of 200 μm, while the feeder channel has a width of 700 µm in the vicinity of the junction.

**Figure 5:**
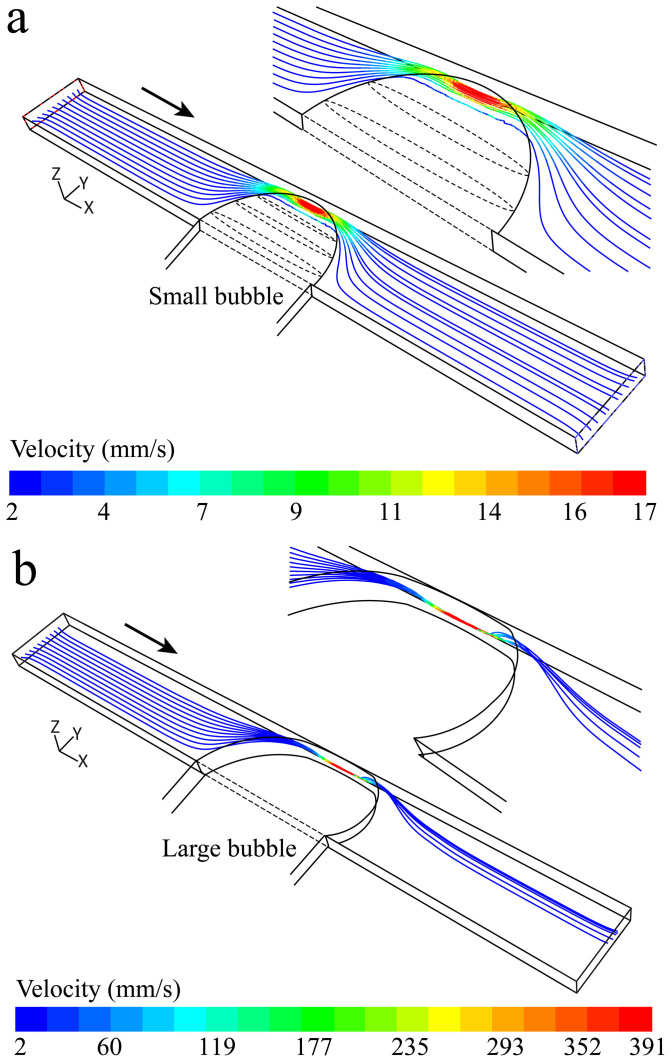
Trajectory of 15 µm particles passing through the channel obtained by CFD simulations: (a) For the case of small bubble shown in Figure 4b, the particles are not orderly patterned downstream of the bubble, (b) For the case of a large bubble shown in Figure 4e, the particles have the tendency to orderly pattern along a narrow band downstream of the bubble.

Further computational fluid dynamics (CFD) simulations are carried out to study the trajectory of suspended particles at the downstream of the bubbles using ANSYS Fluent 6.3 software (Canonsburg, PA, USA). For comparison, the two extreme cases shown in Figures 4b and 4e, are investigated. The top-view geometry of the bubbles is adopted from these figures, while the cross-sectional geometry of the bubbles is shown in the inset of Figures 5a and 5b. The flow patterns are obtained by solving the Navier-Stokes equations through the channel by assuming steady state and laminar conditions^35^. Next, the trajectory of particles is obtained using the Lagrangian particle tracking model^39^ as follows:
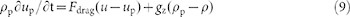
where, *ρ*_p_ and *ρ* are the densities of the particle and the liquid, respectively, *u* and *u*_p_ are the local velocities of the liquid and particle, respectively, and *g*_z_ is the gravitational acceleration. *F*_drag_ is the hydrodynamic drag term, which is defined as 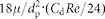
40, where *d*_p_ is the diameter of the particle, *Re* is the relative Reynolds number defined as 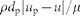, and *C*_d_ is the drag coefficient, as defined by Morsi *et* *al*.^40^.

The flow rate of the liquid at the outlet is set to 2 µL/min. A group of ten 15 µm particles are released from the inlet surface at a height of 30 µm with respect to the bottom surface of the channel. For the case of Figure 5a, the particles can pass through the gap *via* different patterns depending on their initial location along the width of the channel. This also enables multiple particles to pass through the gap at the same time. The particles are slightly displaced compared to their initial locations but do not follow any ordered patterns, as observed in Figure 4b.

Alternatively, for the case of Figure 5b, the gap between the bubble and the opposite sidewall is so small that only one particle can pass through the gap at a time. At the downstream of the bubble, the particles are deflected towards the middle of the channel until the inertial, gravitational, pressure and shear forces reach a balance. The simulations indicate that the particles are focused along a narrow band with a width of ∼ 45 µm, which is slightly higher than that of observed in Figure 4e. This difference can be attributed to several factors, including the smaller gap between the bubble and the opposite sidewall, or the distortion of the flow by the passing particles and the deformation of particles, which are both ignored in the numerical model.

## Discussion

In summary, we developed a hydrodynamically actuated bubble-based microfluidic system. This microfluidic system consists of a high-resolution hydrodynamic bubble actuator for inserting and controlling bubbles *via* axillary feeder channels. The system precisely moves the bubbles along the feeder channels to dynamically alter the characteristics of flow within the main channel (Figure 1). An active micromixer was achieved by oscillating the bubble within the feeder channel at desired frequencies and displacements to disturb the neighbouring streams (Figure 2). A microvalve was demonstrated by changing the size of the bubble to partially or fully close the main channel (Figure 3). Furthermore, the hydrodynamic patterning of suspended particles was obtained by creating a large bubble within the main channel and changing the flow streamlines (Figure 4). The designed system is versatile and its characteristics could be tightly controlled *via* tuning the bubble actuations and displacements. Such reconfigurable systems can reduce the time and effort needed for multiple unit re-design and re-fabrication, enabling universal systems that can be used for a variety of applications. The systems were fabricated by single-layer photolithography, relied on low-cost mechanical hardware, and had relatively simple operational procedures.

In contrast to laser-assisted generation and actuation of bubbles, our strategy does not affect the temperature of the surrounding medium^30^,^31^, and thus is suitable for bio processes in integrated emerging lab-on-a-chip devices^41^. Moreover, our approach does not use any high power optical sources, and therefore do not require additional safety procedures. However, the response time of the bubbles in the current work is in the order of a fraction of second, which is still much longer than that of laser-assisted systems with response times in the micro second order^29^.

Compared to electrolytic or thermo-pneumatic actuation of bubbles, our strategy does not require patterning of metallic electrodes^21^ or resistive heaters^26^ onto the glass substrates to control the size of the bubbles, which in turn reduces the time and cost of fabrication. Additionally, its operation is not limited to ionic liquid as of electrolytic systems.

Advantageously, our strategy does not require the entrapment of bubbles during the filling process of the microfluidic chip^23^,^26^,^27^, as the air pocket is stored at the tip of the feeder tube, and injected into the feeder channel using the bubble actuator.

Unlike pneumatic actuation of bubbles, our strategy does not need additional air supplies, pressure controllers or valves^25^. Instead, it takes advantage of hydrodynamic bubble actuators, which are in principle high-resolution small, inexpensive and easy to operate syringe pumps. However, with respect to scalability, the pneumatic actuated systems are superior to the presented system^25^.

In comparison to acoustic actuation of bubbles, which can only be used in a dynamic mode to oscillate the bubbles^23^, the hydrodynamic actuation of bubbles enables the displacement of the bubbles in both static and dynamic modes. In static mode, the bubbles can maintain their dimensions, geometries and locations for extended periods of time that allow the realisation of a tightly controlled bubble-based microvalve concept (Figure 3). Alternatively in dynamic modes, the bubbles characteristics can be rapidly tuned to enable bubble-based micromixers (Figure 2). Altogether, such examples suggest that the same bubble-based system can serve as a multi-functional actuator for achieving a variety of functionalities using a single unit. Although, there are previous demonstration of multi-functional platforms capable of pumping, mixing, and controlling of flow, such systems have so far relied on multi-layer microfluidics for including pneumatic membrane actuation^42^. In contrast, the presented system can be fabricated by single-layer photolithography processes. More importantly, the hydrodynamic actuation of the bubbles does not rely on any soft membrane deformation. This means that our system can be used for establishing glass, silicon, SU-8 or poly methyl-methacrylate (PMMA) based microfluidics.

We anticipate the hydrodynamically actuated bubble-based systems to find extensive range of applications in microfluidics as their offer simple fabrication and operation process, as well as versatility. This strategy is bio-compatible, not sensitive to electrochemical properties of the buffer, and can be operated at low voltages using portable batteries, enabling integration into lab-on-a-chip systems. While we have demonstrated the capabilities of our bubble-based system in a basic microfluidic system comprising a main channel, it has the potential to be implemented in more complicated microfluidic units comprising a network of channels. Several bubbles can be operated to realise multiple functions at a time and also to improve the efficiency of the system for specific functions such as mixing. The system can also be improved by the integration of image analysis feedback modules, making it fully automated.

## Methods

The hydrodynamic actuator utilises a micro-controller (Arduino Uno SMD, ATMEGA328), a high-resolution stepper-motor (Nema 17, Hybrid 0.9 degree stepper motor) with 400 steps per revolution (SPR), a custom-made mechanical gear to transform rotational motion into linear motion with a linear pitch of *P*_shaft _ = 0.525 mm, a 1 ml glass syringe (Becton) with an internal diameter of 4.64 mm, a Tygon® tube with an inner diameter of 1/32 inch to connect the syringe to the microfluidic chip. More detail is given in Supplementary Information-1. The syringe, feeder tube and the feeder channel are in series, and the displacement of the water column or air bubble can be estimated as below:
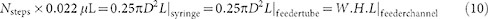
where *N*_steps_ is the number of steps rotated by the stepper-motor, *D* is the diameter, *L* is the displacement, *W* is the width and *H* is the height.

The microchannel was fabricated from polydimethylsiloxane (PDMS) using standard soft lithography and replica molding techniques. To create the PDMS structure, PDMS base and curing agent (Sylgard 184, Dow Corning Corporation, MI) were mixed with a ratio of 10:1 *w*/*w* and degassed in an vacuum oven until all the bubbles disappeared.

A syringe pump (Harvard Apparatus, PHD2000, USA) is employed to withdraw the liquid through the outlet of the main channel at desired flow rates. The performance of the microfluidic system is monitored using an inverted microscope (Nikon, Eclipse TE2000) equipped with a camera (Basler A102fc, Germany).

The response time of the bubbles is measured by monitoring the displacement of the air bubble through the feeder channel using an inverted microscope (Nikon Eclipse Ti), which is equipped with an electron-multiplying CCD camera (QuantEM 512SC) capable of capturing images at a rate of 50 frames per second, as detailed in **Figure** **S4**.

The snapshots of Supplementary Movies 2 to 6 are given in Supplementary Information 3.

## Author Contributions

A.A. and H.A. fabricated the bubble-based actuator system, performed experiments, and evaluated the data. R.S. analysed the data. P.Y. fabricated the microfluidic chips. K.K. designed experiments, and performed analytical and numerical calculations. K.K. and K.K.Z. wrote the manuscript. K.K. and K.K.Z. coordinated and supervised the work.

## Supplementary Material

Supplementary Information Supplementary Information

Supplementary Information Supplementary movie 1

Supplementary Information Supplementary movie 2

Supplementary Information Supplementary movie 3

Supplementary Information Supplementary movie 4

Supplementary Information Supplementary movie 5

Supplementary Information Supplementary movie 6
