# Corrigendum: A multi-functional bubble-based microfluidic system

**DOI:** 10.1038/srep46950

**Published:** 2018-03-01

**Authors:** Khashayar Khoshmanesh, Abdullah Almansouri, Hamad Albloushi, Pyshar Yi, Rebecca Soffe, Kourosh Kalantar-zadeh

Scientific Reports 5: Article number: 994210.1038/srep09942; published online: 04 23 2015; updated: 03 01 2018

The Acknowledgements section in this Article was omitted. The Acknowledgements section should read:

“Khashayar Khoshmanesh acknowledges the Australian Research Council for funding under the Discovery Early Career Researcher Award (DECRA) scheme (Project No. DE120101402).”

