# Supplementary material for: A multi-functional bubble-based microfluidic system
Source: Sci Rep. 2015 Apr 23;5:9942. doi: 10.1038/srep09942 (PMC4407724; doi:10.1038/srep09942)
Supplement: Supplementary Information [file srep09942-s1.pdf]

Supplementary Information:

A Multi-functional Bubble-based Microfluidic System

Khashayar Khoshmanesh [†], *, Abdullah Almansouri [†], Hamad Albloushi [†], Pyshar Yi, Rebecca Soffe, and Kourosh Kalantar-zadeh *

School of Electrical and Computer Engineering, RMIT University, Melbourne, Victoria 3001, Australia

* Corresponding authors:

khashayar.khoshmanesh@rmit.edu.au

kourosh.kalantar@rmit.edu.au

[†] These authors have equal contributions

Supplementary Informaion-1: Technical details of the bubble-based microfluidic system

a. Experimental setup

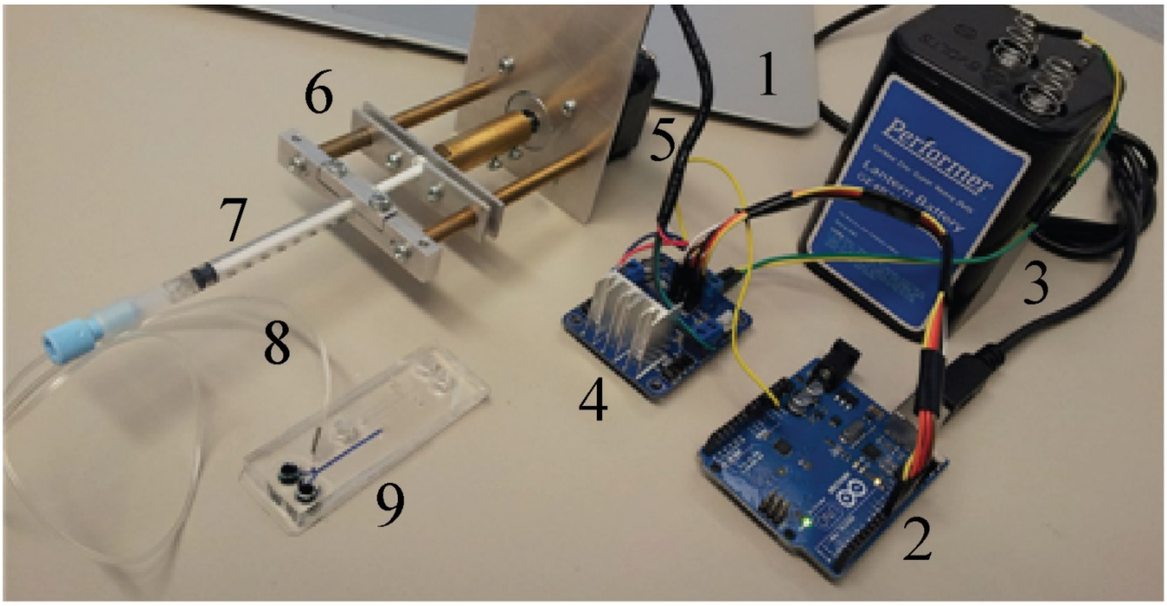

Figure S1. The details of the bubble-based microfluidic system, comprising of: (1) computer to run a MATLAB based code to command the microcontroller, (2) micro-controller (Arduino Uno SMD, ATMEGA328), (3) 6 V battery, (4) stepper-motor driver, (5) high-resolution stepper-motor (Nema 17, Hybrid 0.9 degree stepper motor) with 400 steps per revolution (SPR), (6) custom-made mechanical gear to transform rotational motion into linear motion with a linear pitch of P_{shaft} = 0.525 mm, (7) 1 ml glass syringe (Becton) with an internal diameter of 4.64 mm, (8) feeder tube (Tygon® tube with an internal diameter of 1/32 inch), and (8) microfluidic chip.

b. Mechanical gear

The mechanical gear utilises a motor shaft as the internal thread (female), and a screw as the external thread (male). By rotating the motor shaft, the screw can push or pull the plunger of the syringe. The pitch of the screw is 0.525 mm, and hence the minimum linear displacement for the mechanical gear is 1.31 μm.

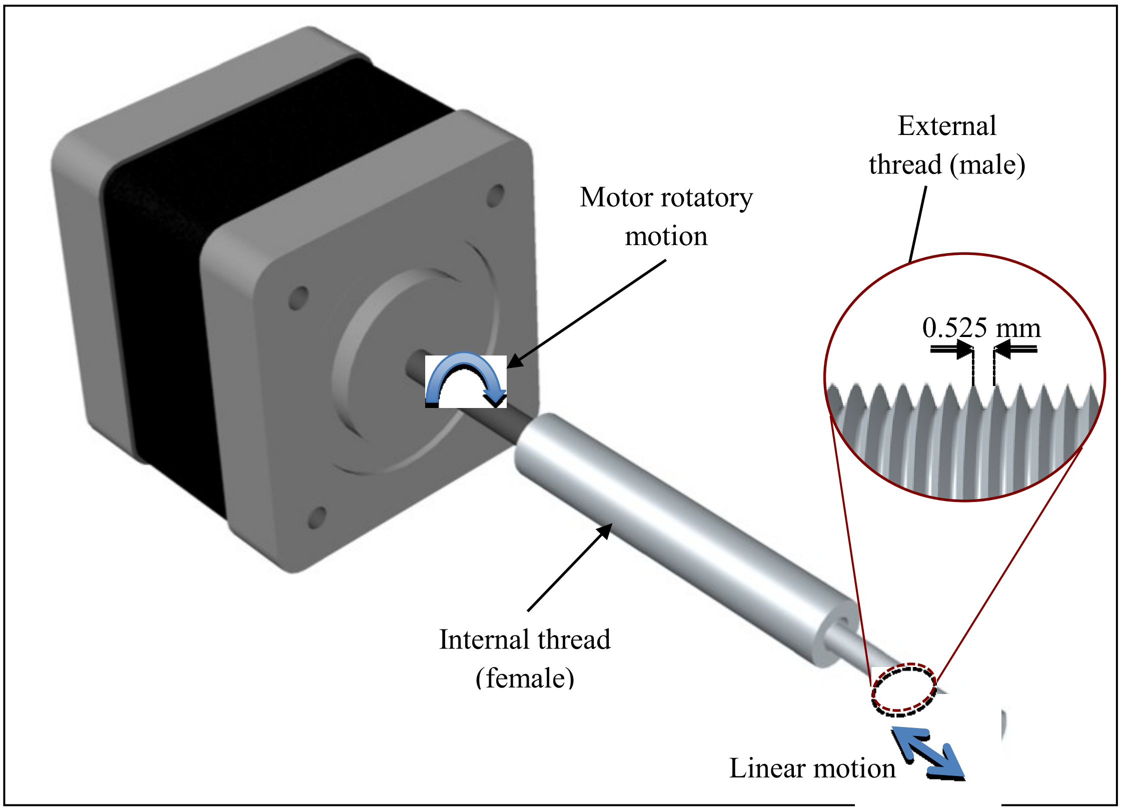

Figure S2. Schematics of high-resolution custom-made bubble actuator: The Nema 17 stepper motor shaft connected to a female thread, which has a pitch of 0.525 mm. The rotation of the motor pushes the screw away or toward the motor.

c. Electrical interface

Arduino UNO SMD board is utilised as the electronic interface between the computer and the stepper-motor, as it is inexpensive, small in size, and has a good clock speed (16 MHz). The board is connected to the computer using a USB connection. The other end of the Arduino is connected to a high-voltage, high-current dual H-bridge integrated circuit (L298n). The chip is used as the driver of the stepper-motor. **Figure S3** shows the schematics of the system.

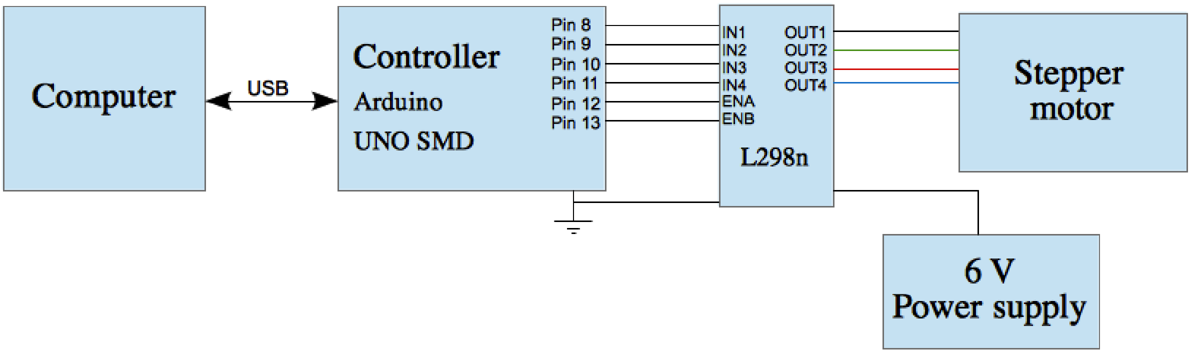

Figure S3. Functional block diagram for the electrical interface of the high-resolution custom-made bubble actuator.

d. MATLAB code

The software has been developed in the MATLAB environment to enable the user from controlling the mechanical hardware. The software allows the user to rotate the motor in one direction by specified number of steps (1 step = 0.9 degree) and with the desired speed. It also enables the user to continuously alternate the direction of rotation (clockwise and anti-clockwise). **Figure S4** shows the interface of the software.

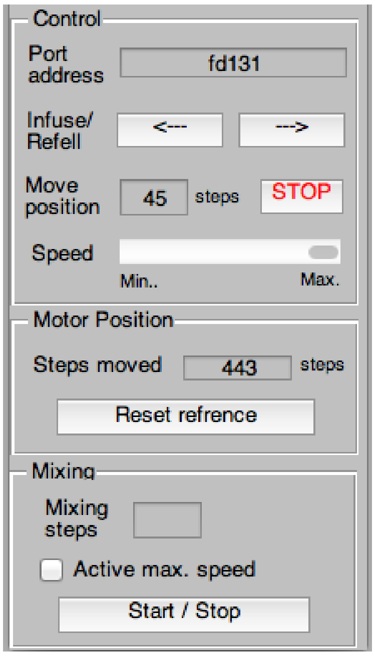

Figure S4. MATLAB GUI to control the stepper-motor (courtesy of MathWorks).

Supplementary Informaion-2: Measuring the response time of bubbles

In order to measure the response time of the bubble, we compare the variations of the sinusoidal actuation signal applied to the mechanical gear with the displacement of the bubble tip along the feeder channel when oscillated at a displacement of 1095 μm with a frequency of 1.22 Hz. In doing so, we monitor the displacement of the bubble using an inverted microscope (Nikon Eclipse Ti) equipped with an electron-multiplying CCD camera (QuantEM 512SC), which is capable of acquiring images at a rate of 50 frames per second. The images are post-processed using Matlab's image processing toolbox (MathWorks, 2010a) to track the tip of the bubble. **Figure S5** shows the normalized variations of the applied signal and the displacement of the bubble tip with respect to time in 3 cycles. The results indicate a short delay of < 20 ms when the bubble is moving towards the sidewall while an elongated delay of up to 80 ms when the bubble is moving away from the sidewall.

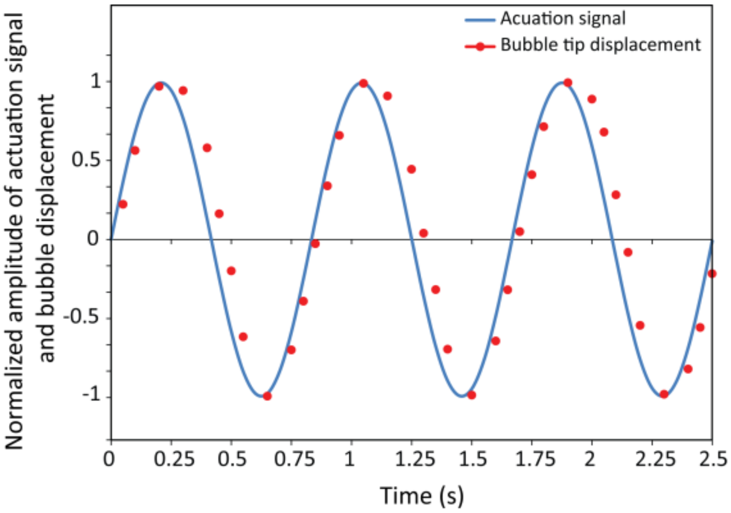

Figure S5. Normalized variations of the applied signal and the displacement of the bubble tip with respect to time in 3 cycles.

Supplementary Informaion-3: Utilisation of bubble-based micromixer in smaller microfluidic channels

To ensure the hydrodynamic actuation of bubbles can be implemented to smaller microfluidic systems, we scaled down the system shown in **Figure 2** by a factor of 40%. The width of the main channel and the feeder channel junction were reduced to 200 and 300 µm, respectively as shown below. The bubble was oscillated at a displacement of 250 µm and a frequency of 2.5 Hz, while a flow rate of 0.8 µL/min was provided through the main channel. Under these settings, the average velocity of oscillating bubble along the feeder channel and the average velocity of the flow within the main channel were maintained at 1250 µm/s and 0.9 mm/s, respectively, to be consistent with the case of the larger microfluidic system, presented in **Figure 2**. This resulted in a mixing efficiency of 63±3% at a distance of 800 µm away from the feeder channel junction, which was comparable to the results obtained with the larger microfluidic system.

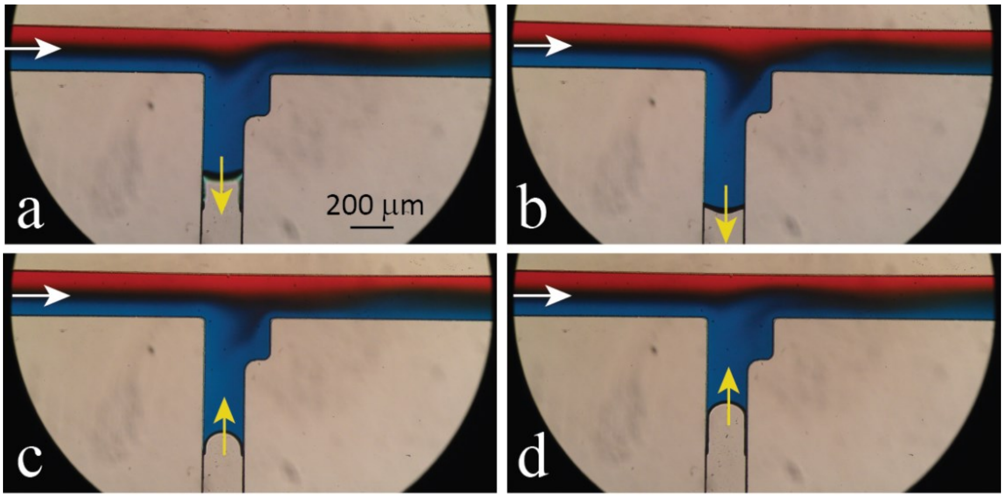

Figure S6. Utilisation of the bubble-based micromixer in a small microfluidic channel with a width of 200 µm. The bubble was oscillated at a displacement of 250 µm and with a frequency of 2.5 Hz, while a flow rate of 0.8 µL/min was provided through the main channel.

Supplementary Informaion-4: Snapshots of Supplementary Movies 2-6

Supplementary movie 2-Part 1:

Flow rate = 2 μL/min, Bubble displacement = 767 μm, Frequency = 1.56 Hz

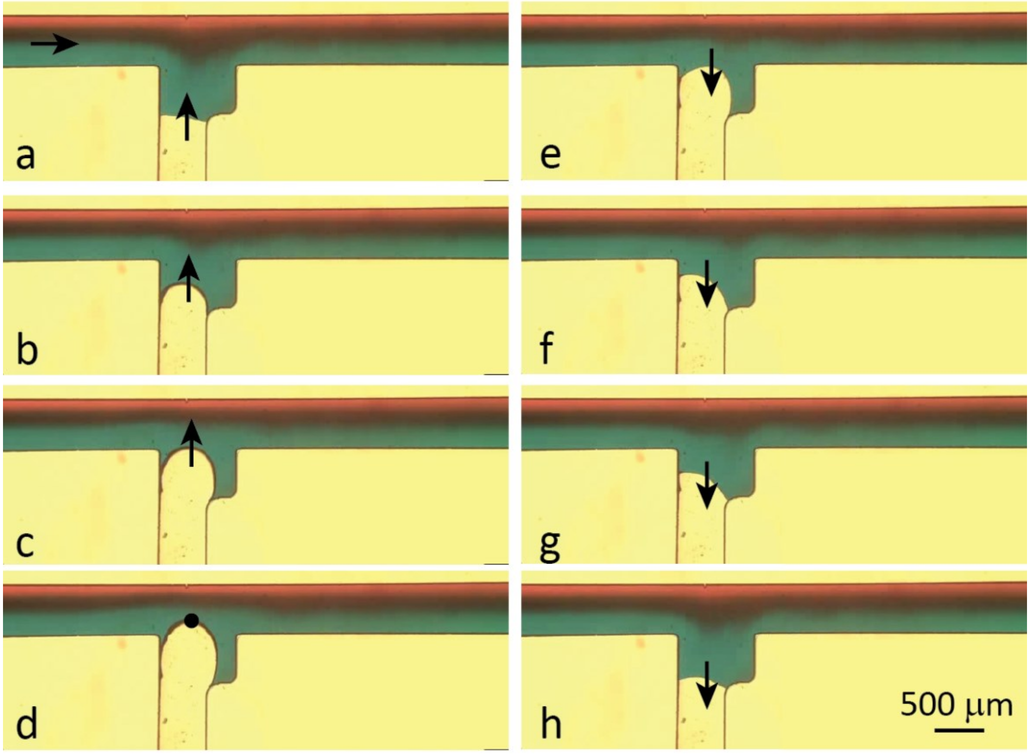

Figure S7. Snapshots of Supplementary Movie 2-Part 1: The operation of the bubble-based micromixer by oscillating the bubble along the feeder channel. The bubble displacement is 767 μm and the bubble oscillation frequency is 1.56 Hz. The flow rate of the water moving through the main channel is 2 μL/min.

Supplementary movie 2-Part 2:

Flow rate = 2 μL/min, Bubble displacement = 1095 μm, Frequency = 1.2 Hz

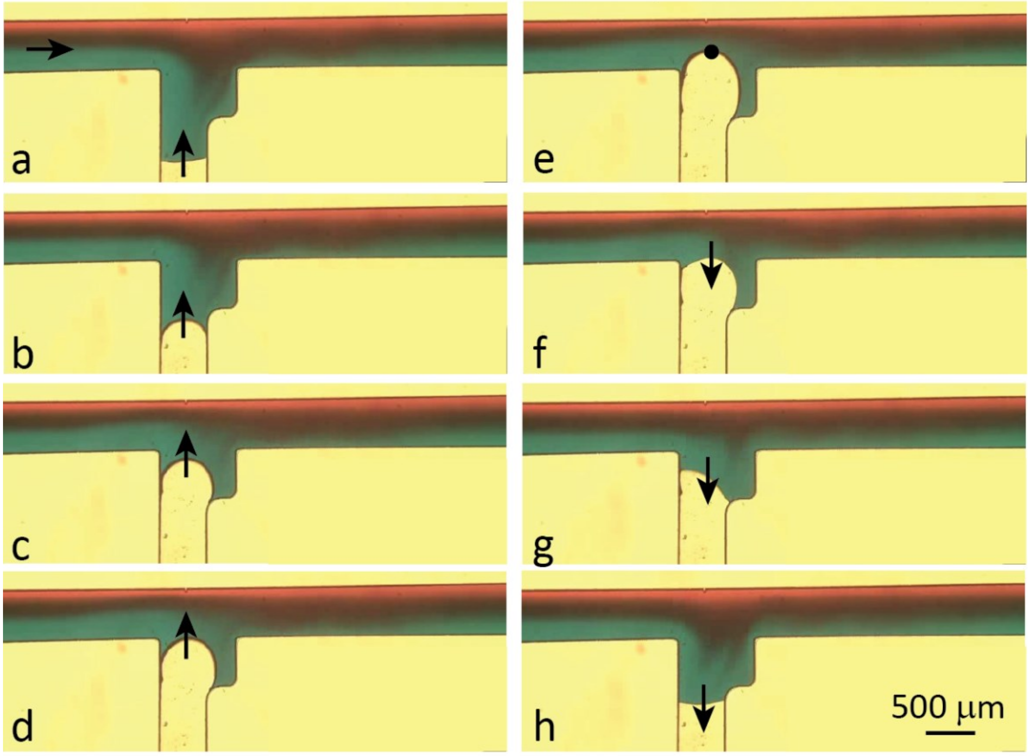

Figure S8. Snapshots of Supplementary Movie 2-Part 2: The operation of the bubble-based micromixer by oscillating the bubble along the feeder channel. The bubble displacement is 1095 μm and the bubble oscillation frequency is 1.2 Hz. The flow rate of the water moving through the main channel is 2 μL/min.

Supplementary movie 3:

Flow rate = 5 μL/min, Bubble displacement = 1209 μm, Frequency = 0.99 Hz

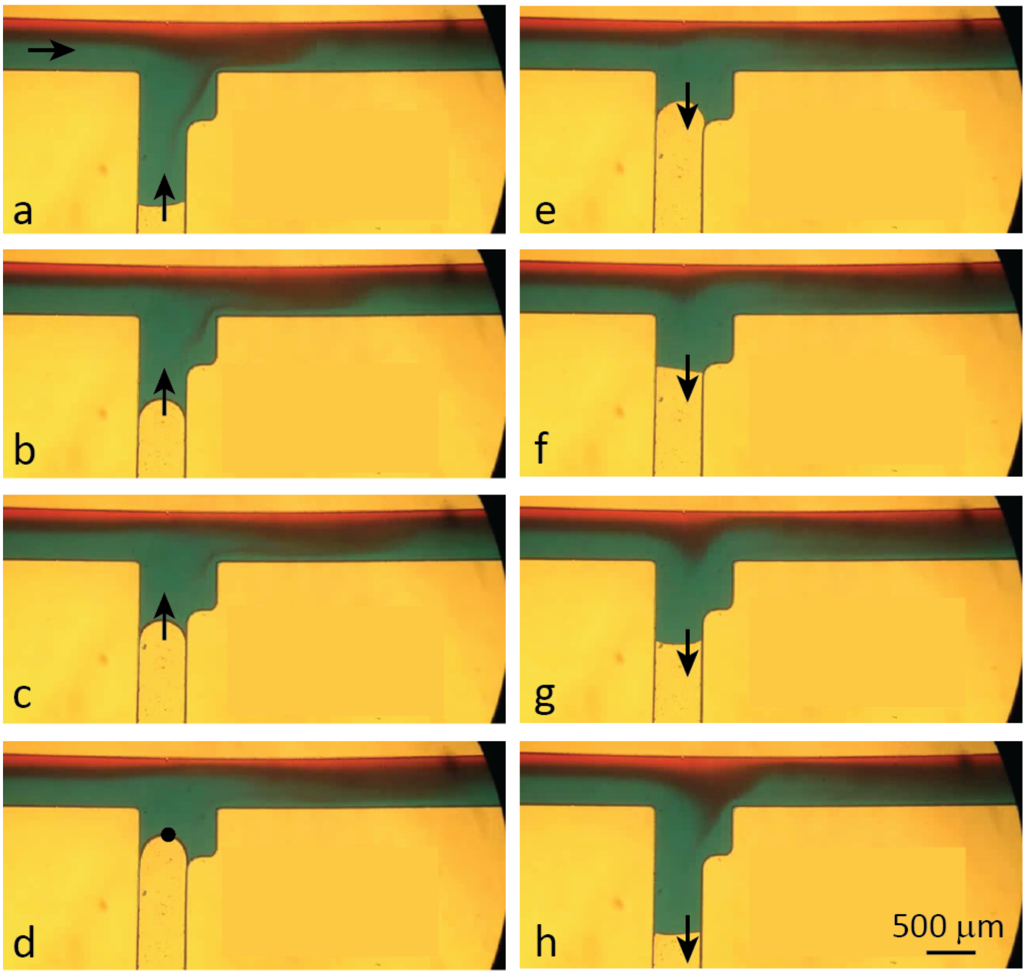

Figure S9. Snapshots of Supplementary Movie 3: The operation of the bubble-based micromixer by oscillating the bubble along the feeder channel. The bubble displacement is 1209 μm and the bubble oscillation frequency is 0.99 Hz. The flow rate of the water moving through the main channel is 5 μL/min.

Supplementary movie 4:
Flow rate = 10 μL/min

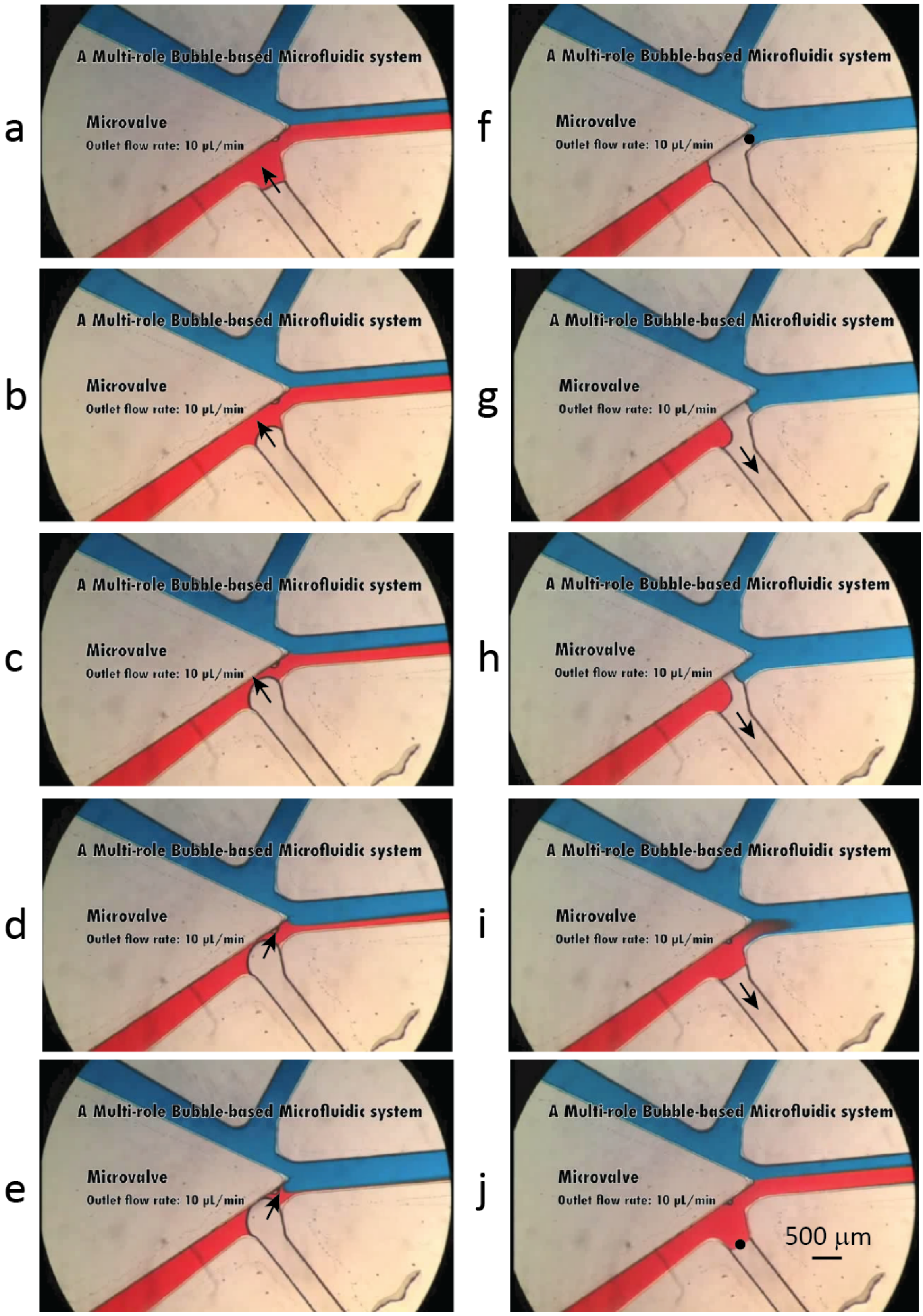

Figure S10. Snapshots of Supplementary Movie 4: The operation of the bubble-based microvalve by changing the location of the bubble along the feeder channel. The flow rate of the water moving through the main channel is 10 μL/min.

Supplementary movie 5:

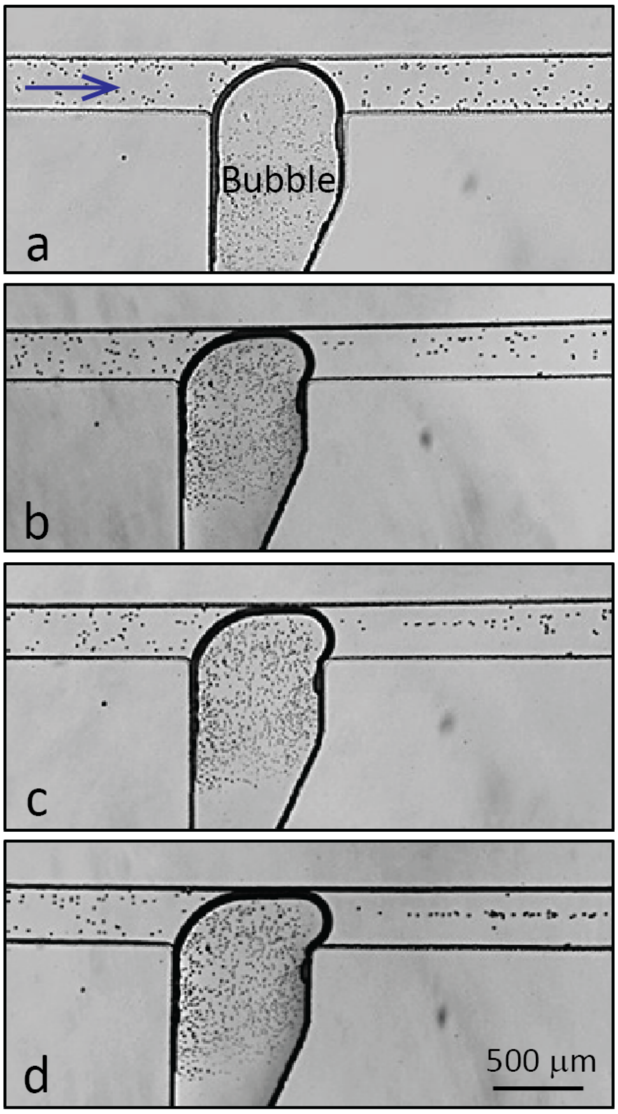

Figure S11. Snapshots of Supplementary Movie 5: Hydrodynamic patterning of 15 μm polystyrene particles by creating a large bubble along the main channel. The flow rate of the water moving through the main channel is 2 μL/min.

Supplementary movie 6:

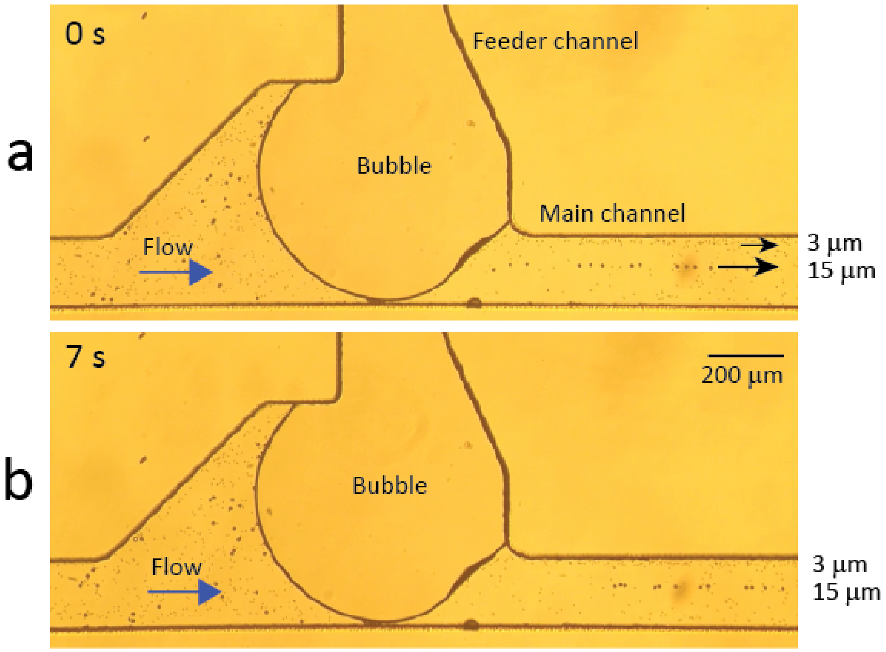

Figure S12. Snapshots of Supplementary Movie 6: Demonstrating the size-based separation of polystyrene particles obtained by creating a large bubble along the main channel. The main channel has a width of 200 µm while the feeder channel has a width of 700 µm in the vicinity of the junction.
